# Supplementary material for: Water Stress Alters Morphophysiological, Grain Quality and Vegetation Indices of Soybean Cultivars
Source: Plants (Basel). 2022 Feb 21;11(4):559. doi: 10.3390/plants11040559 (PMC8880803; doi:10.3390/plants11040559)
Supplement: Supplementary file 1 [file plants-11-00559-s001.zip › Table S1 .pdf]

Table S1. Mean values of vegetation indices, gas exchange and photosystem II maximum quantum yield in six soybean cultivars under four water regimes from 2018 and 2019.

Cultivars	WR	Variables														
		NDVI	SAVI	DVI	GNDVI	NDRE	TCARI	OSAVI	TO	PRI	*A*	g_s	iWUE	*E*	Fv/Fm	CI
BRS 5980IPRO	31	0.34	0.23	0.14	0.55	0.14	0.16	0.28	0.64	0.30	4.5	0.05	90.7	2.1	0.79	232
	44	0.41	0.27	0.17	0.58	0.18	0.17	0.34	0.58	0.32	10.0	0.11	90.9	2.6	0.78	216
	64	0.46	0.31	0.19	0.60	0.20	0.19	0.38	0.51	0.32	17.8	0.36	49.4	5.3	0.81	261
	100	0.51	0.34	0.20	0.61	0.22	0.21	0.42	0.58	0.33	19.7	0.35	56.3	5.0	0.81	256
BRS 7180IPRO	31	0.42	0.30	0.19	0.58	0.19	0.18	0.35	0.66	0.33	3.7	0.06	61.7	2.6	0.75	241
	44	0.49	0.36	0.24	0.61	0.23	0.21	0.43	0.61	0.35	10.9	0.18	60.6	3.5	0.78	226
	64	0.57	0.41	0.27	0.62	0.25	0.26	0.49	0.55	0.35	18.3	0.33	55.5	5.3	0.80	249
	100	0.76	0.53	0.34	0.69	0.31	0.33	0.64	0.52	0.34	19.9	0.36	55.3	5.1	0.82	254
BRS 7280RR	31	0.39	0.26	0.16	0.58	0.16	0.17	0.32	0.63	0.30	10.0	0.07	142.9	2.3	0.79	184
	44	0.44	0.30	0.19	0.60	0.20	0.18	0.37	0.58	0.34	15.3	0.15	102.0	3.2	0.80	204
	64	0.50	0.35	0.22	0.61	0.22	0.22	0.43	0.53	0.33	20.7	0.33	62.7	5.2	0.82	248
	100	0.56	0.38	0.23	0.62	0.23	0.25	0.47	0.58	0.32	21.2	0.35	60.6	5.2	0.83	251
BRS 7380RR	31	0.37	0.26	0.16	0.55	0.17	0.17	0.31	0.68	0.31	5.3	0.10	53.0	2.4	0.78	188
	44	0.44	0.31	0.20	0.58	0.20	0.19	0.38	0.64	0.35	12.7	0.17	74.7	3.5	0.80	217
	64	0.48	0.34	0.22	0.59	0.21	0.22	0.41	0.57	0.34	18.8	0.33	57.0	5.1	0.80	257
	100	0.64	0.44	0.28	0.61	0.24	0.32	0.54	0.65	0.34	20.7	0.36	57.5	5.1	0.81	250
M 6410IPRO	31	0.39	0.26	0.16	0.56	0.17	0.16	0.33	0.61	0.30	6.9	0.07	98.6	2.2	0.78	283
	44	0.45	0.30	0.19	0.60	0.22	0.17	0.37	0.56	0.34	13.9	0.18	77.2	2.9	0.79	216
	64	0.49	0.34	0.21	0.61	0.23	0.19	0.41	0.50	0.34	19.0	0.29	65.5	4.6	0.82	256
	100	0.55	0.38	0.23	0.63	0.25	0.22	0.46	0.56	0.34	20.9	0.36	58.1	5.2	0.82	251
NA 5909GR	31	0.34	0.22	0.13	0.56	0.15	0.15	0.28	0.60	0.29	7.2	0.06	120.0	3.6	0.80	233
	44	0.41	0.27	0.16	0.59	0.19	0.16	0.34	0.56	0.33	14.0	0.15	93.3	3.2	0.82	222
	64	0.48	0.32	0.19	0.61	0.21	0.20	0.39	0.54	0.34	20.4	0.31	65.8	4.7	0.83	251
	100	0.53	0.35	0.21	0.63	0.23	0.21	0.43	0.55	0.34	21.1	0.36	58.6	5.2	0.84	255
Mean		0.33	0.20	0.60	0.21	0.20	0.40	0.58	0.33	4.0	15.9	0.22	72.2	4.0	0.80	237
SE		0.02	0.01	0.01	0.01	0.01	0.01	0.02	0.01	0.01	0.68	0.03	0.22	0.2	0.01	4.94
CV		2.03	2.13	2.29	0.52	1.84	2.33	2.07	0.84	0.52	1.86	5.67	3.2	3.0	2.19	1.02

WR: water regimes (31, 44, 64 and 100% CET replacement - WR1, WR2, WR3, and WR4 respectively); NDVI: Difference Vegetation Index; SAVI: Soil Adjusted Vegetation Index; DVI: Difference Vegetation Index; GNDVI: Green Normalized Difference Vegetation Index; NDRE: Red Edge Normalized Difference; TCARI: The Transformed Chlorophyll Absorption and Reflectance Index; OSAVI: Optimized Soil Adjusted Vegetation Index; TO: TCARI/OSAVI Ratio; PRI: Photochemical Reflectance Index; A: net assimilation of CO_2 (μmol CO_2 m^{-2} s^{-1}); g_s: stomatal conductance (μmol H_2O m^{-2} s^{-1}); iWUE: intrisic water use efficiency [A: net assimilation of CO_2 (μmol CO_2 m^{-2} s^{-1})/g_s: stomatal conductance (μmol H_2O m^{-2} s^{-1})]; E: transpiration (mmol H_2O m^{-2} s^{-1}); Fv/Fm: photosystem II Maximum Quantum Yield; CI: Internal Carbon Concentration;SE: standard error; CV: coefficient of variation (%).
