# Supplementary material for: Water Stress Alters Morphophysiological, Grain Quality and Vegetation Indices of Soybean Cultivars
Source: Plants (Basel). 2022 Feb 21;11(4):559. doi: 10.3390/plants11040559 (PMC8880803; doi:10.3390/plants11040559)
Supplement: Supplementary file 1 [file plants-11-00559-s001.zip › Table S2.pdf]

Table S2. Mean values of morphological evaluations, grain quality and yield in six soybean cultivars under four water regimes from 2018 and 2019.

Cultivars	WR	Variables														
		A1	PH	SD	NK	PP	GP	GM	HW	MTG	GY	G	WUE	O	P	D
BRS5980IPRO	31	1.03	25.8	4.7	10.1	18.9	2.06	10.0	63.10	142.8	2338	73.3	11.20	19.1	39.5	23.0
	44	0.96	28.4	4.9	11.6	26.3	2.01	11.1	65.78	141.9	2782	92.6	8.82	19.1	39.6	2.2
	64	0.83	29.3	5.0	11.5	32.4	2.10	11.8	63.15	141.8	3271	92.3	7.20	19.0	39.0	1.0
	100	0.84	30.1	5.2	12.1	36.1	2.08	11.8	64.33	151.4	3351	93.3	4.85	19.6	38.8	0.3
BRS7180IPRO	31	1.16	35.4	2.7	9.9	22.2	1.98	11.6	61.00	134.0	2419	88.6	9.83	21.4	38.7	5.5
	44	0.98	38.8	5.9	12.2	29.8	2.10	10.7	63.24	152.2	3026	82.0	7.05	19.7	38.5	2.3
	64	1.01	38.9	6.4	11.6	34.3	2.26	11.5	64.70	162.3	3305	69.0	5.45	20.4	37.3	4.7
	100	0.90	42.2	6.3	11.2	39.5	2.09	13.5	63.07	168.5	3431	54.0	4.08	21.3	37.0	0.2
BRS7280RR	31	1.13	39.9	6.0	11.3	19.3	2.08	10.4	64.70	178.1	3117	86.0	12.03	20.3	40.4	6.2
	44	1.07	36.1	6.0	10.9	28.7	2.17	11.0	63.10	180.2	3628	92.0	8.82	20.7	39.1	2.2
	64	1.13	43.5	5.6	14.5	26.5	2.28	11.1	63.80	203.1	4312	87.3	7.28	19.6	39.8	0.0
	100	1.04	41.5	5.5	11.8	29.7	2.13	11.1	63.72	212.8	4342	87.0	4.86	21.0	38.9	0.7
BRS7380RR	31	1.06	32.5	5.1	11.6	21.2	2.18	11.5	61.10	133.2	2527	93.6	10.48	19.9	39.7	0.7
	44	0.93	35.5	5.5	12.8	30.6	2.18	11.7	61.07	141.3	3052	91.3	7.90	21.3	38.0	0.2
	64	0.96	37.4	5.6	13.1	36.2	2.20	11.9	62.32	158.0	3474	83.3	5.96	22.1	38.0	0.0
	100	0.95	37.4	5.8	13.9	47.6	2.16	11.9	62.00	173.7	3751	87.3	4.36	22.1	37.6	0.0
M6410IPRO	31	0.99	32.0	4.9	11.1	17.6	2.09	10.8	62.02	139.1	2604	83.3	10.91	20.6	40.3	14.5
	44	0.94	32.1	4.9	11.1	23.0	2.25	11.2	62.32	148.1	3286	95.6	8.48	20.8	38.9	1.2
	64	0.95	34.3	5.1	12.4	30.8	2.22	11.8	61.60	165.4	3544	90.0	6.18	22.9	36.7	0.0
	100	0.88	35.2	5.0	12.0	33.2	2.08	11.8	63.98	183.3	4013	86.0	4.40	22.6	36.3	0.0
NA5909GR	31	0.91	25.5	6.7	10.6	19.9	2.05	10.1	62.83	169.7	2910	76.0	11.66	21.9	37.5	19.8
	44	0.85	28.2	5.7	11.0	27.4	1.86	11.3	63.93	167.6	3419	94.6	8.52	21.1	37.9	1.5
	64	0.87	30.0	5.6	12.1	29.9	1.87	11.5	63.73	192.5	4055	90.3	6.82	21.4	37.5	0.2
	100	0.81	31.6	6.1	12.6	41.1	1.82	11.4	65.95	205.4	4261	88.6	4.82	21.8	37.5	1.8
Mean		0.96	34.2	6.7	11.80	29.3	2.10	11.3	63.19	164.4	3342	85.7	7.40	20.8	38.4	38.4
SE		0.02	1.04	0.93	0.22	1.56	0.02	0.15	0.28	4.75	119.46	1.94	1.02	0.23	0.23	0.23
CV		1.01	1.49	6.76	0.92	2.60	0.58	0.63	0.22	1.41	1.75	1.11	3.48	0.54	0.30	0.30

WR: water regimes (31, 44, 64 and 100% CET replacement - WR1, WR2, WR3, and WR4 respectively); A1: height of first pod set (cm); PH: plant height (cm); SD: stem diameter (mm); NK: number of knots; PP: number of pods; GP: grains per pod; GM: grain moisture (%); HW: hectoliter weight; MTG: mass of thousand grains (g); GY: grain yield (kg ha^{-1}); G: germination (%); WUE: water use efficiency; O: oil (%); P: protein (%); D: hard seeds (%); SE: standard error; CV: coefficient of variation (%).
